# Carbapenem Nonsusceptible *Klebsiella pneumoniae* in Taiwan: Dissemination and Increasing Resistance of Carbapenemase Producers During 2012–2015

**DOI:** 10.1038/s41598-018-26691-z

**Published:** 2018-05-31

**Authors:** Sheng-Kang Chiu, Ling Ma, Ming-Chin Chan, Yi-Tsung Lin, Chang-Phone Fung, Tsu-Lan Wu, Yin-Ching Chuang, Po-Liang Lu, Jann-Tay Wang, Jung-Chung Lin, Kuo-Ming Yeh

**Affiliations:** 1 Division of Infectious Diseases and Tropical Medicine, Department of Medicine, Tri-Service General Hospital, National Defense Medical Center, Taipei, Taiwan, ROC; 2 Institute of Infectious Diseases and Vaccinology, National Health Research Institutes, Miaoli, Taiwan, ROC; 3 Infection Control Office, Tri-Service General Hospital, National Defense Medical Center, Taipei, Taiwan, ROC; 4 Section of Infectious Diseases, Department of Medicine, Taipei Veterans General Hospital, National Yan-Ming University, Taipei, Taiwan, ROC; 5 Division of Infectious Diseases, Department of Internal Medicine, Sijhih Cathy General Hospital, New Taipei City, Taiwan, ROC; 6 Department of Laboratory Medicine, Chang Gung Memorial Hospital, Kweishan, Taoyuan, Taiwan, ROC; 7 Department of Medical Research, Chi Mei Medical Center, Tainan, Taiwan, ROC; 8 Department of Internal Medicine, Kaohsiung Medical University Hospital, Kaohsiung, Taiwan, ROC; 9 Division of Infectious Diseases, Department of Medicine, National Taiwan University Hospital, Taipei, Taiwan, ROC

## Abstract

Before 2011, the prevalence rates of carbapenemase-producing *Klebsiella pneumoniae* (CPKP) among carbapenem nonsusceptible *K*. *pneumoniae* (CnSKP) isolates were below 10% in Taiwan. The study presents the dissemination and increased antimicrobial resistance of CPKP from January 2012 to August 2015, as shown by Taiwanese multicenter surveillance. Isolates with minimum inhibitory concentrations (MICs) of >1 μg/mL for imipenem or meropenem were collected, screened for various carbapenemase genes by PCR, and tested for antimicrobial susceptibility. Among 1,457 CnSKP isolates, 1,250 were collected from medical centers. The CnSKP prevalence in medical centers increased by 1.7-fold during the study. Among all CnSKP isolates, 457 were CPKP. The CPKP rate among CnSKP increased by 1.5-fold and reached 36.8% in 2015. The CPKP nonsusceptibility rate to aztreonam, fluoroquinolones, and aminoglycosides increased yearly. Six CPKP isolates carried dual carbapenemase genes. Three Ambler classes were identified in 451 isolates with a single carbapenemase: classes A (315 *bla*_KPC-2_, 2 *bla*_KPC-3_, 28 *bla*_KPC-17_, 2 *bla*_KPC-34_), B (26 *bla*_IMP-8_, 2 *bla*_NDM-1_, 36 *bla*_VIM-1_), and D (40 *bla*_OXA-48_). The *bla*_OXA-48_ rate among CPKP increased by 6-fold over three years. Most KPC and OXA-48 producers were ST11. CnSKP was increasingly prevalent, owing to CPKP dissemination. Additionally, CPKP became more resistant during the study period.

## Introduction

*Klebsiella pneumoniae* is a common cause of bacteremia, pneumonia, urinary tract infection, and liver abscess^1^. β-lactam antibiotics are often deemed the primary therapeutic option for these infections^2^. Among the β-lactams, carbapenems are considered the antibiotics of last resort^3^. Once *K*. *pneumoniae* isolates become nonsusceptible to carbapenems, they are often resistant to all currently available β-lactams and frequently resistant to non-β-lactam antibiotics^4^. In the clinical context, the emergence of carbapenem non-susceptible *K*. *pneumoniae* (CnSKP) poses a serious threat to patient survival because CnSKP infections have limited treatment options and are associated with high mortality^5^.

Among the many mechanisms conferring resistance to carbapenems, carbapenemases can efficiently hydrolyze carbapenems and have become an important cause of antimicrobial resistance^6^. Many carbapenemases have been identified in *K*. *pneumoniae* and are classified into Ambler class A (KPC, GES, IMI, NMC, SME), class B (IMP, VIM, NDM, GIM, SIM, SPM), and class D (OXA-48)^7^.

The molecular epidemiology of carbapenemase-producing *K*. *pneumoniae* (CPKP) varies by country. In the United States, *K*. *pneumoniae* carbapenemase-producing *K*. *pneumoniae* (KPC-KP) was first reported in 2001^8^ and subsequently caused outbreaks in New York City^9^. KPC has become the predominant carbapenemase in the U.S.^10^ and China^11^. OXA-48 was first identified in *K*. *pneumoniae* in Turkey in 2003^12^ and is the major carbapenemase in Turkey^13^ and Spain^14^. NDM-KP was first detected in 2008 in a patient returning to Sweden from India^15^ and is the most common CPKP in India^16^.

In Taiwan, IMP-8 was the first reported carbapenemase in *K*. *pneumoniae* in 2001^17^. Our previous study revealed that IMP-8 was the only type of carbapenemase in CPKP prior to 2009^18^. In a national surveillance study in 2010, we identified VIM-1 from *K*. *pneumoniae* isolates^4^. In the same year, *K*. *pneumoniae* isolates producing NDM-1 and KPC-2 were identified in Taiwanese patients returning from India and China, respectively^19,20^. Subsequently, outbreaks caused by KPC-KP were reported in northern Taiwan in 2011^21^.

After the arrival of KPC-2 producers, the molecular epidemiology and antimicrobial resistance profile of CnSKP and CPKP in Taiwan has not been examined well. Herein, we analyzed these trends from 2012 to 2015 to better understand and possibly prevent their global spread.

## Results

### Prevalence of CnSKP and Specimen Sources

A total of 1,457 CnSKP isolates were collected during the study period. Among all CnSKP, 1,250 (85.8%) were collected from medical centers, and 207 (14.2%) from regional hospitals. In three major regional hospitals, 137 isolates nonsusceptible to carbapenem were identified from 6,446 clinical *K*. *pneumoniae* isolates during the study period, and the average prevalence of CnSKP was 2.13%.

**Figure 1:**
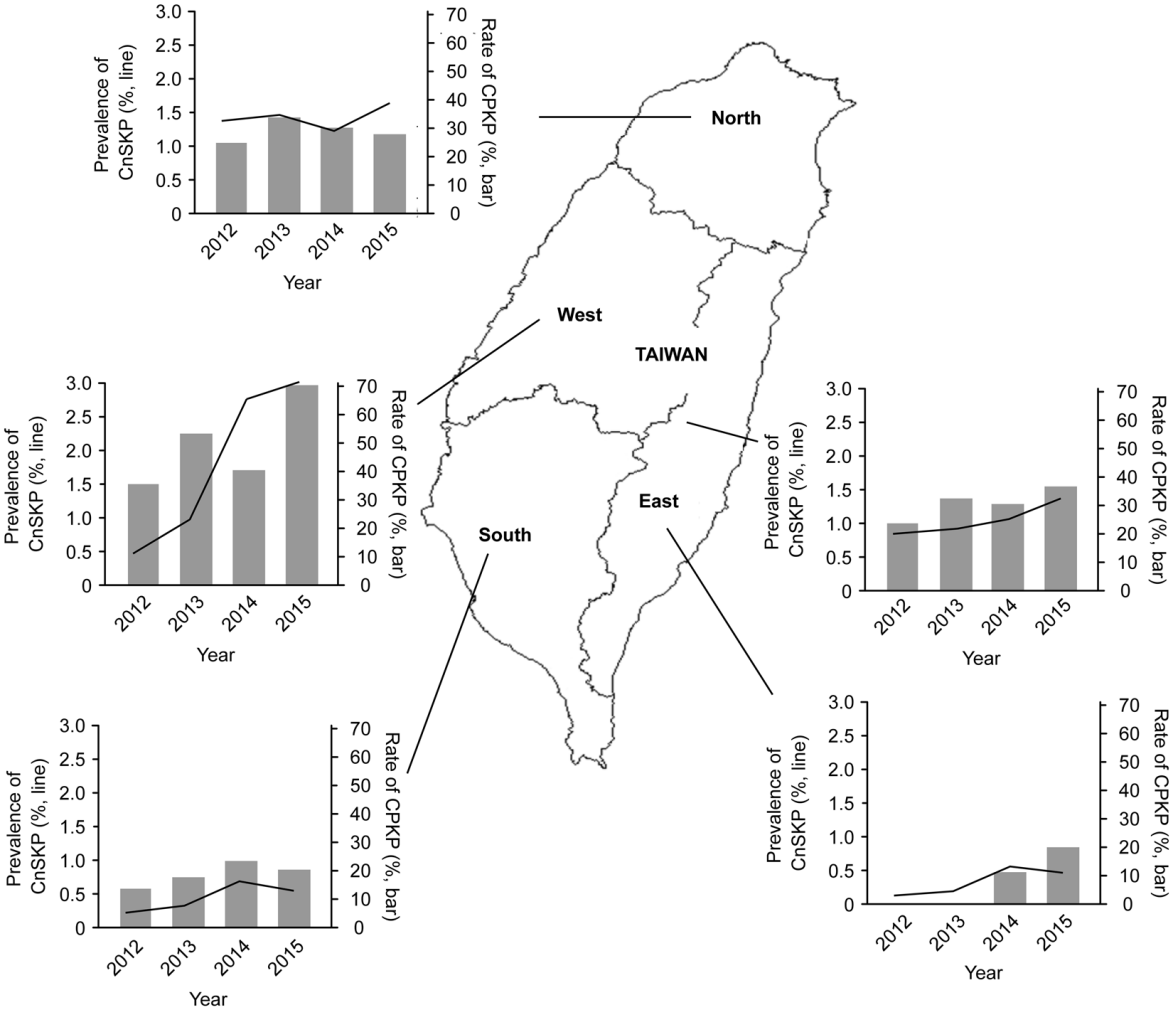
Temporal trends in the prevalence of carbapenem nonsusceptible *Klebsiella pneumoniae* (CnSKP) among clinical isolates in major medical centers (line) and the rate of carbapenemase-producing isolates (CPKP) among CnSKP (bar) by geographic region in Taiwan from 2012 to 2015. The graphs in the figure are original graphs, and the map was created using CorelDRAW Graphics Suite X4 software.

In all medical centers, the average prevalence of CnSKP was 1.10%, with 114,065 clinical isolates screened during the study period. The temporal trends in prevalence were analyzed by geographic region (Fig. 1). In the North region, the average prevalence was 1.40%, with no significant increase during the study period (p = 0.380). In the West region, the prevalence significantly increased (p < 0.001) 6-fold from 0.50% (14/2778) in 2012 to 3.12% (91/2917) in 2015, which was the highest yearly prevalence observed by region in Taiwan. The prevalence also increased significantly in the South and East regions (p < 0.001 and p = 0.040, respectively). Overall, the prevalence of CnSKP in Taiwan significantly increased 1.7-fold (0.87% in 2012, 1.46% in 2015; p < 0.001).

CnSKP isolates were recovered primarily from urine (518, 35.6%), followed by sputum (including bronchoalveolar lavage; 461, 31.6%), abscess/drainage (158, 10.8%), blood (130, 8.9%), bile (38, 2.6%), ascites (30, 2.1%), and central vein catheters (28, 1.9%). The rates for blood and abscess/drainage increased significantly yearly (6.3%, 9.1%, 8.3%, 11.5%, p = 0.040; 8.7%, 8.0%, 12.5%, 13.2%, p = 0.016; respectively). The rate of bacteremia increased nearly 2-fold within the 4 years studied. The isolation rates for sputum and central vein catheters significantly decreased annually (36.4%, 35.1%, 26.7%, 30.8%, p = 0.024 and 3.1%, 2.3%, 1.5%, 1.1%, p = 0.043, respectively). The specimen sources of CnSKP are summarized in Table 1.

**Table 1:** Specimen sources of the carbapenem nonsusceptible *Klebsiella pneumoniae* isolates collected from 21 hospitals in Taiwan during 2012 and 2015.

	Total (N = 1457)	Specimen source (n, %)				*p* value*
		2012 (N = 286)	2013 (N = 350)	2014 (N = 457)	2015 (N = 364)	
Abscess/Drainage	158 (10.8)	25 (8.7)	28 (8.0)	57 (12.5)	48 (13.2)	**0.016**
Ascites	30 (2.1)	5 (1.7)	9 (2.6)	8 (1.8)	8 (2.2)	0.932
Bile	38 (2.6)	6 (2.1)	9 (2.6)	13 (2.8)	10 (2.7)	0.583
Blood	130 (8.9)	18 (6.3)	32 (9.1)	38 (8.3)	42 (11.5)	**0.040**
Central vein catheter	28 (1.9)	9 (3.1)	8 (2.3)	7 (1.5)	4 (1.1)	**0.043**
Rectal swab/Stool	48 (3.3)	0 (0.0)	0 (0.0)	27 (5.9)	21 (5.8)	**<0.001**
Sputum**	461 (31.6)	104 (36.4)	123 (35.1)	122 (26.7)	112 (30.8)	**0.024**
Urine	518 (35.6)	113 (39.5)	112 (32.0)	183 (40.0)	110 (30.2)	0.127
Other***	46 (3.2)	6 (2.1)	29 (8.3)	2 (0.4)	9 (2.5)	**0.043**

*Chi-square for trend.

**Including specimens from bronchoaveolar lavage.

***Including pleural effusion, synovial fluid, throat swab, and vaginal discharge.

### Temporal Trends of CPKP

Of the 1,457 CnSKP isolates, 457 (31.4%) were positive for genes encoding carbapenemase (CPKP), while no carbapenemase genes were detected in 1,000 isolates (non-CP-CnSKP). The temporal trends for the rate of CPKP among CnSKP were analyzed by geographic region (Fig. 1). In the West region, the yearly rate of CPKP significantly increased nearly 2-fold from 35.7% to 70.3% in four years (p = 0.002). The rates in the North, South and East regions also increased, but not significantly (p = 0.695, 0.447, 0.570; respectively). Overall, the rate of CPKP in Taiwan significantly increased 1.5-fold from 24.1% to 36.8% during the study period (p = 0.003). The rate of non-CP-CnSKP decreased from 75.9% to 63.2%.

### Antimicrobial Nonsusceptibility Rates

All CnSKP isolates were tested with β-lactam and non-β-lactam agents (Table 2). For the β-lactams, most (>99%) of the CnSKP isolates were nonsusceptible to cephalosporins, except for cefepime (nonsusceptible rate, 92.2%). The rate of nonsusceptibility to cefepime was lowest among non-carbapenem β-lactams, and the rates to meropenem and doripenem were the lowest among all β-lactams.

**Table 2:** Antimicrobial nonsusceptibility rates of carbapenem non-susceptible *Klebsiella pneumonia* (CnSKP), carbapenemase-producing *K*. *pneumoniae* (CPKP) and non-CP-CnSKP isolates collected in Taiwan from 2012 to 2015.

	Non-susceptibility (no. %)			*p* value	OR	95% CI
	CnSKP (n = 1457)	CPKP (n = 457)	Non-CP-CnSKP (n = 1000)			
TZP	1410 (96.8)	449 (98.2)	961 (96.1)	**0.036**	2.278	1.056–4.914
CFZ	1457 (100.0)	457 (100.0)	1000 (100.0)	n/a	n/a	n/a
FOX	1453 (99.7)	456 (99.8)	997 (99.7)	0.784	1.372	0.142–13.227
CXM	1457 (100.0)	457 (100.0)	1000 (100.0)	n/a	n/a	n/a
CTX	1455 (99.9)	456 (99.8)	999 (99.9)	0.580	0.456	0.028–7.314
CAZ	1455 (99.9)	456 (99.8)	999 (99.9)	0.580	0.456	0.028–7.314
FEP	1343 (92.2)	455 (99.6)	888 (88.8)	**<0.001**	28.694	7.056–116.683
DOR	1170 (80.3)	454 (99.3)	716 (71.6)	**<0.001**	60.026	19.128–188.366
ETP	1393 (95.6)	456 (99.8)	937 (93.7)	**0.001**	30.660	4.239–221.754
IPM	1436 (98.6)	457 (100.0)	979 (97.9)	n/a	n/a	n/a
MEM	1160 (79.6)	448 (98.0)	712 (71.2)	**<0.001**	20.135	10.263–39.502
ATM	1404 (96.4)	442 (96.7)	962 (96.2)	0.625	1.164	0.634–2.138
CIP	1326 (91.0)	430 (94.1)	896 (89.6)	**0.006**	1.849	1.193–2.866
LVX	1287 (88.3)	421 (92.1)	866 (86.6)	**0.003**	1.810	1.230–2.662
GEN	841 (57.7)	210 (46.0)	631 (63.1)	**<0.001**	0.497	0.397–0.622
AMK	408 (28.0)	97 (21.2)	311 (31.1)	**<0.001**	0.597	0.460–0.775
SXT	1213 (83.3)	324 (70.9)	889 (88.9)	**<0.001**	0.304	0.229–0.403
CST	214 (14.7)	66 (14.4)	148 (14.8)	0.858	0.972	0.710–1.330
TGC	180 (12.4)	52 (11.4)	128 (12.8)	0.298	0.748	0.432–1.293

Abbreviations: AMK, amikacin; ATM, aztreonam; CAZ, ceftazidime; CFZ, cefazolin; CIP, ciprofloxacin; CST, colistin; CTX, cefotaxime; CXM, cefuroxime; DOR, doripenem; ETP, ertapenem; FEP, cefepime; FOX, cefoxitin; GEN, gentamicin; IPM, imipenem; LVX, levofloxacin; MEM, meropenem; n/a, not applicable SXT, trimethoprim-sulfamethoxazole; TGC, tigecycline; TZP, piperacillin-tazobactam. *Comparison between CPKP and non-CP-CnSKP with univariate logistic regression. Statistics that are significant are written in bold font.

For the non-β-lactams, the nonsusceptibility rates of CnSKP to the fluoroquinolones (91.0% to ciprofloxacin and 88.3% to levofloxacin) were similar to that of cefepime. Among all tested agents, the rate of nonsusceptibility to tigecycline was the lowest, followed by colistin and the aminoglycosides.

The nonsusceptibility rates of CPKP and non-CP-CnSKP isolates were analyzed and compared, and different patterns were observed (Table 2). To β-lactams, CPKP were highly nonsusceptible, with rates >99% to all cephalosporins and >98% to piperacillin-tazobactam and all carbapenems. Aztreonam showed the lowest rate among all β-lactams. While non-CP-CnSKP isolates showed similar rates to most cephalosporins and aztreonam, they were significantly less nonsusceptible to piperacillin-tazobactam, cefepime, and carbapenems than their counterparts. The most obvious difference between these two groups were the rates of nonsusceptibility to doripenem and meropenem, which for non-CP-CnSKP were only 71.6% and 71.2%, respectively.

Among the non-β-lactams, CPKP isolates were significantly more nonsusceptible to fluoroquinolones than non-CP-CnSKP. No significant difference was seen between the two groups in terms of nonsusceptibility to colistin and tigecycline.

### Temporal Trends of Nonsusceptibility Rates

**Figure 2:**
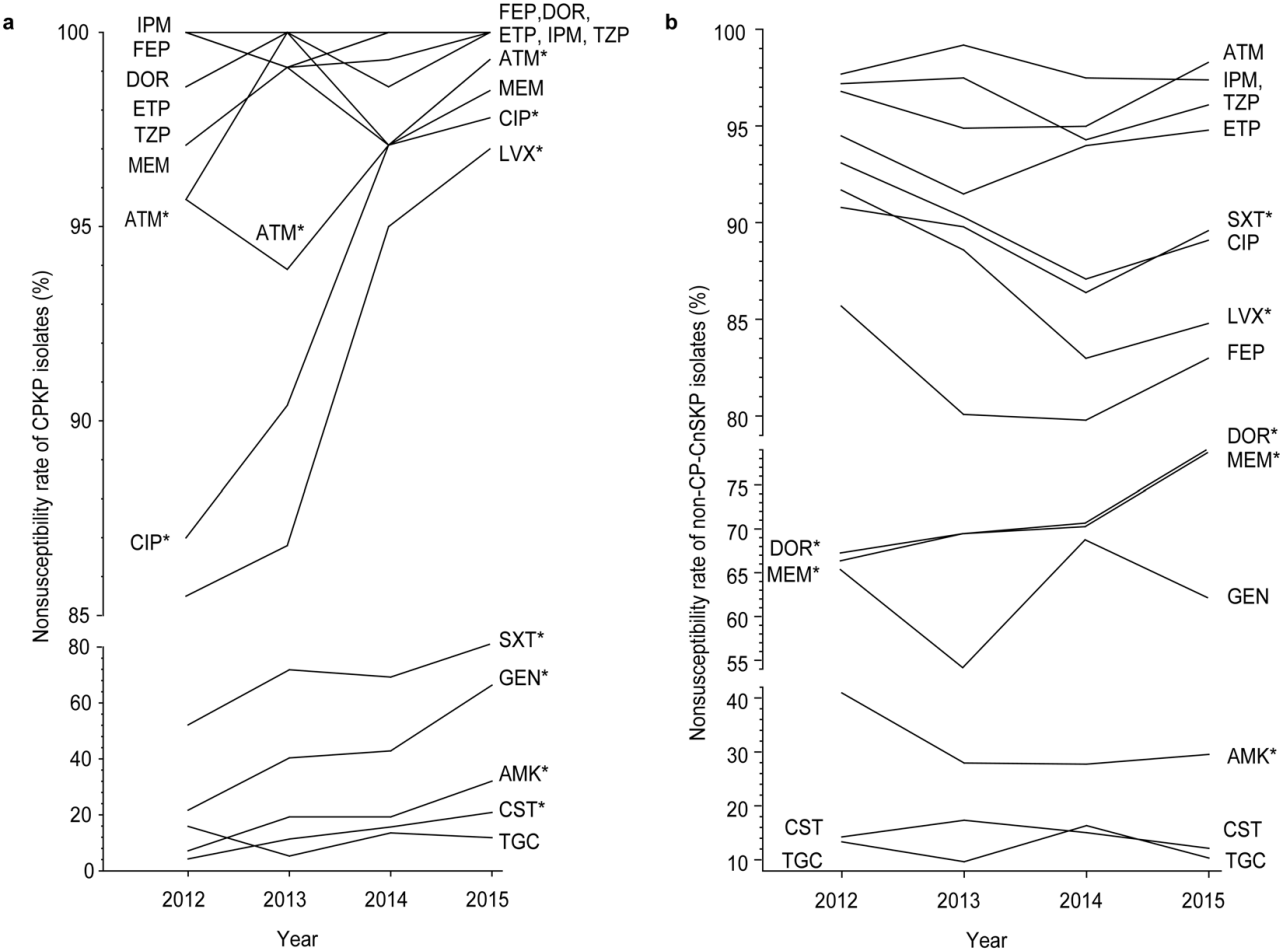
Temporal antimicrobial nonsusceptibility rates of (**a**) carbapenemase-producing *Klebsiella pneumoniae* (CPKP) and (**b**) noncarbapenemase-producing carbapenem-nonsusceptible *K*. *pneumoniae* (non-CP-CnSKP) collected in Taiwan from 2012 to 2015. Abbreviations: AMK, amikacin; ATM, aztreonam; CAZ, ceftazidime; CIP, ciprofloxacin; CST, colistin; CTX, cefotaxime; DOR, doripenem; ETP, ertapenem; FEP, cefepime; FOX, cefoxitin; GEN, gentamicin; IPM, imipenem; LVX, levofloxacin; MEM, meropenem; SXT, trimethoprim-sulfamethoxazole; TGC, tigecycline; TZP, piperacillin-tazobactam. *p < 0.05, chi-square test for trend.

The yearly trends for the nonsusceptibility rates of CPKP and non-CP-CnSKP isolates were analyzed (Fig. 2a,b, respectively). While being the least nonsusceptible β-lactam for CPKP, the rate of nonsusceptibility to aztreonam significantly increased during the study period (p = 0.040). A similar trend was also observed in non-β-lactams. The rates of nonsusceptibility to fluoroquinolones and trimethoprim-sulfamethoxazole also increased significantly (all p < 0.001). The rates of nonsusceptibility to gentamicin, amikacin, and colistin significantly increased 3- (21.7% to 66.4%), 4- (7.2% to 32.1%), and 5-fold (4.3% to 20.9%), respectively (all p ≤ 0.001).

Non-CP-CnSKP isolates showed increasing nonsusceptibility to doripenem and meropenem, with rates that increased from 67.3% to 79.1% (p = 0.007) and from 66.4% to 78.7%, (p = 0.006), respectively; however, the rates of nonsusceptibility to levofloxacin and amikacin decreased significantly (from 91.7% to 84.8%, p = 0.007; and from 41.0% to 29.6%, p = 0.012; respectively).

### Carbapenemase Types and Temporal Trends of CPKP

Among the 457 CPKP isolates, 6 (1.3%) were positive for dual carbapenemases, including 3 isolates for KPC-2/OXA-48, 2 for NDM-1/VIM-1, and 1 for KPC-2/IMP-8. Among the 451 CPKP isolates with a single carbapenemase, 347 (76.9%) were class A KPC [315 KPC-2, 2 KPC-3, 28 KPC-17, and 2 novel-variant KPC-34 (GenBank accession no. KU985429)], 64 (14.2%) were class B MBL (26 IMP-8, 2 NDM-1, and 36 VIM-1), and 40 (8.9%) were class D OXA-48. No matching hits were detected for GES, IMI, NMC, SME, GIM, SIM, or SPM.

**Figure 3:**
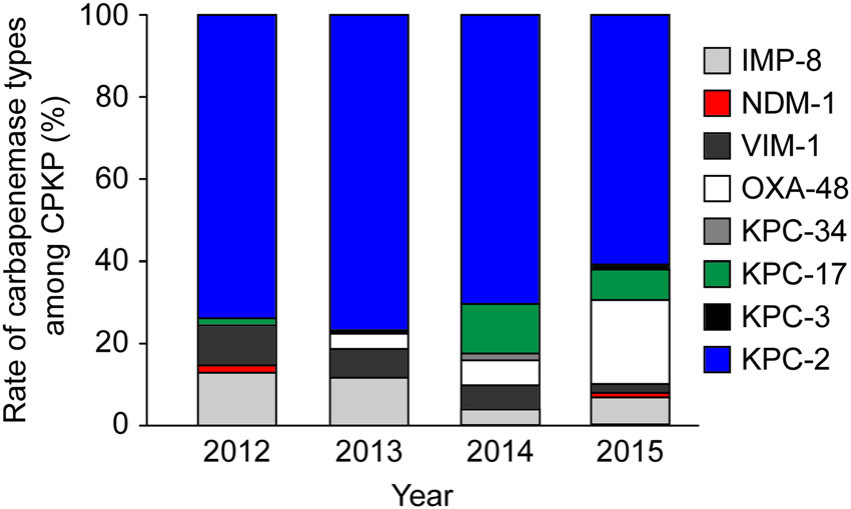
Temporal trends in the rates of various subgroups among carbapenemase-producing *Klebsiella pneumoniae* (CPKP) in Taiwan from 2012 to 2015.

The temporal trends of the rate of carbapenemase types among the 451 CPKP with a single carbapenemase were analyzed (Fig. 3). While KPC-2 was predominant throughout the study period, the rate of KPC-2 producers decreased significantly from 73.9% (51/69) in 2012 to 61.1% (80/131) in 2015 (p = 0.011). KPC-17 producers were first identified in 2012, and their rate increased significantly during the study period (p = 0.005). While only 4 (3.5%) isolates with the OXA-48 gene were found in 2013, this rate significantly increased nearly 6-fold to 20.6% (27/131) within three years (p < 0.001). The rates of IMP-8 and VIM-1 producers decreased significantly (p = 0.015 and p = 0.031, respectively), and KPC-3, KPC-34, and NDM-1 producers were identified sporadically.

### Multilocus Sequence Types of CPKP

All CPKP were characterized with MLST. Among those with dual carbapenemases, all of the 3 KPC-2/OXA-48 producers were ST11, both of the NDM-1/VIM-1 producers were ST273, and the KPC-2/IMP-8 producer was ST11.

Among those with a single carbapenemase, most (340/347, 98.0%) of the KPC-KP were ST-11, including all KPC-17, both KPC-34, 309 (98.1%) KPC-2 and 1 (50%) KPC-3. ST11 was predominant among OXA-48 producers as well, with 87.5% (35/40) being ST11. MBL producers, however, were diverse in MLST; more than eight MLSTs (ST-11, -37, -45, -225, -309, -1087, -1192, -1355) were identified in IMP-8 producers, and only one IMP-8 was ST11. More than 11 MLSTs (ST-12, -29, -34, -268, -273, -307, -327, -656, -681, -741, -1665) were identified among VIM-1-producing isolates, but none was ST11. Of NDM-1 producers, one was ST11 and the other was ST313.

In summary, four CPKP among the six isolates with dual carbapenemases were ST11 (4/6, 66.7%) and 377 CPKP among the 451 isolates with single carbapenemase were ST11 (377/451, 83.6%).

## Discussion

In this surveillance study, the prevalence of CnSKP in Taiwanese medical centers averaged 1.10% and increased 1.7-fold during the study period. This prevalence was much lower than that reported in the U.S. (6%), according to a report from the 2007–2009 SENTRY Antimicrobial Surveillance Program^10^. A similar trend of increased CnSKP prevalence during the same period in Taiwan was observed by the Taiwan Nosocomial Infections Surveillance (TNIS) which only included those isolates collected from patients in intensive care units. The prevalence from the TNIS was indeed much higher than that in this study, in which we collected isolates from all patients, regardless they were inpatients or outpatients. The TNIS showed that the prevalence of CnSKP in intensive care units in Taiwanese medical centers increased nearly 1.5-fold from 15.7% in 2012 to 22.5% in 2015^22^. The geographic differences of CnSKP prevalence of medical centers found in this study were also reported by the TNIS, with the highest prevalence being 28.1% in the West region in 2015^22^. Geographic differences in CnSKP prevalence were also reported in the U.S.^23^. While an increase of 1.7-fold over four years in this study is not so impressive, the prevalence of CnSKP in the TNIS increased 15.7-fold from 1.8% in 2005 to 28.2% in 2017^22^. Additionally, this study showed that the rates of CPKP and non-CP-CnSKP among CnSKP increased and decreased annually, respectively. The increasing prevalence of CnSKP is regarded as a result of CPKP dissemination.

This study found that the majority of CnSKP in Taiwan were isolated from sputum and urine, which is compatible with other surveys^24,25^. Our study also showed that the incidence of bacteremia increased nearly 2-fold within the four years studied. A similar increase in the incidence of bacteremia caused by CnSKP was also observed in a large teaching hospital in northern Italy^26^. Although *K*. *pneumoniae* is the main cause of liver abscess in Taiwan, those isolates are usually community-acquired and rarely reported to be resistant^1^. However, liver abscess caused by carbapenem-resistant *Klebsiella pneumoniae* was reported in a liver transplant patient in Italy^27^. Moreover, 1.2% (2/165) of *K*. *pneumoniae* isolates causing liver abscess were resistant to carbapenem in a China hospital during 2010–2014^28^. The barrier between community-acquired liver abscess caused by susceptible *K*. *pneumoniae* and hospital-acquired infection by resistant isolates is becoming blurred as resistance is becoming increasingly prevalent.

The rate of CPKP among CnSKP increased annually in Taiwan. A similar trend was also observed in other surveys, which showed a rate of 3.1% between 2010 and 2012^24^ that increased to 20.0% in 2013^25^. While the highest rate in Taiwan was 70.3% in the West region in 2015, rates as high as 89.9% (113/126) and 86.7% (65/75) of CnSKP isolates were identified as CPKP in the U.S.^10^ and Romania^29^, respectively.

Most of the CnSKP in this study were nonsusceptible to all β-lactams and fluoroquinolones; however, some non-β-lactams with low nonsusceptibility rates, such as tigecycline^30^, colistin^31^, and aminoglycosides^32^, may represent potential treatment choices. From an epidemiological study of carbapenem-resistant *Enterobacteriaceae* in U.S.^23^, low nonsusceptibility rates against tigecycline (11.2%), colistin (25.0%) and at least one aminoglycoside (18.3%) were similarly observed.

Different profiles of antimicrobial nonsusceptibility were found between CPKP and non-CP-CnSKP in this study, as CPKP was more nonsusceptible to β-lactams than non-CP-CnSKP. Few studies have compared the resistance profiles between these two groups of clinical isolates; however, a study on carbapenem-resistant *Enterobacteriaceae* demonstrated that carbapenemase-producing isolates were more resistant to meropenem than their non-carbapenemase-producing counterparts^33^.

Our study showed that CPKP was significantly increasingly nonsusceptible to fluoroquinolones and aminoglycosides, while non-CP-CnSKP showed greater nonsusceptibility to doripenem and meropenem, during the study period. Few studies have examined this trend specifically in these resistant bacteria; however, a similar trend for increasing resistance of general *K*. *pneumoniae* isolates was found in national surveillance studies from Taiwan^34^ and Korea^35^.

The coproduction of dual carbapenemases by *K*. *pneumoniae* has been reported sporadically^36,37^. Many combinations have been identified, including KPC/MLB and OXA/MLB^7^; however, the KPC-2/OXA-48 coproducing isolates found in this study have not been reported before to the best of our knowledge. Furthermore, these dual carbapenemase producers belonged to the epidemic ST11 clone. Because of their high resistance profile and transferability, these resistant organisms warrant intensive monitoring.

Since it was first reported in 2001^8^, KPC has spread worldwide^38^. This study showed that KPC outnumbered previous endemic MBL and became the most common carbapenemase in Taiwan soon after its arrival, spreading to all regions of this island. Such rapid dissemination of KPC-KP was also observed in Greece^39^ and Italy^40^. We previously sequenced the complete Taiwanese plasmid encoding KPC-2 and analyzed its high transferability^41^. In this study, we identified one novel *bla*_KPC_ variant, namely, *bla*_KPC-34_, and two that had not been reported in Taiwan before 2012. The first KPC-3 producer was isolated in 2013 and was geographically and genetically linked to those in the U.S.^42^, and the emergence of a KPC-17 producer in Taiwan was also previously observed^25,43^. Similar to the U.S.^10^ and China^11^, KPC was the predominant carbapenemase in Taiwan; however, most of the Taiwanese KPC-producers were ST11, which is similar to China^44^, but different from the U.S., where the dominant clone is ST258^45^. ST11 is a single-locus variant of ST258, indicating their close relationship^46^. However, our previous study showed that *K*. *pneumoniae* ST23 strains are strongly associated with liver abscess in Asian countries^47^. Our study results indicated that these hospital-acquired resistant isolates are quite different from their susceptible counterparts causing community-acquired liver abscess.

After its emergence in 2013, OXA-48 became the second most prevalent type of carbapenemase two years later. Four OXA-48 producers were isolated in 2013 from patients without a travel history abroad^48^. Most of the OXA-48 producers in this study were the endemic ST11 clone, while ST101 was the most prevalent clone in Europe and North Africa^49^. The OXA-48 ST11 clone caused an outbreak in Spain in 2009^47^; however, the plasmids of the Taiwanese and Spanish clones belonged to different incompatibility groups^48,50^.

While this extensive surveillance study involved all regions in Taiwan, not all carbapenemase types were identified during the study period. In a previous study, an isolate producing KPC-16 was reported in the South region in 2014^43^. Additionally, all isolates were screened by PCR in this study, which is consistent with many previous studies^4,24,25^. However, some emerging yet unidentified carbapenemases might be missed by this method. Thus, phenotypic methods, such as Carba NP, the carbapenem inactivation method (CIM), and the modified Hodge test (MHT), are mandatory to identify novel carbapenemases^51^. Moreover, these CnSKP will be assessed using virulence assay, as hypervirulent strains have been reported in China recently^52^.

In conclusion, the current CnSKP, and especially CPKP, scenario in Taiwan is ominous. New types of CPKP have emerged, and these resistant bacteria have become increasingly nonsusceptible to particular antimicrobials, especially aztreonam, fluoroquinolones, trimethoprim-sulfamethoxazole, gentamicin, amikacin, and colistin over time. This increase would make the treatment of infections caused by CnSKP more difficult. Close monitoring and enhanced infection control measures are mandatory to curb the further spread of these highly resistant organisms.

## Materials and Methods

### Study Area and Hospital Settings

Between January 2012 and August 2015, 21 hospitals in Taiwan were enrolled in this study, including 7 in the North, 4 in the West, 7 in the South, and 3 in the East region. Nine of these hospitals are regional hospitals, while 12 are medical centers that provide tertiary care; each of the included hospitals contains more than 1,000 beds. This study was approved by the Institutional Review Boards of participating hospitals, including Chang Gung Memorial Hospital (IRB No.: 1003399B), Taipei Veterans General Hospital (VGHIRB No.: 2011-11-001IC), National Taiwan University Hospital (IRB No.: 20111004 3RB), Tri-Service General Hospital (IRB No.: 100-05-205), Kaohsiung Medical University Chung-Ho Memorial Hospital (KMUH-IRB-20110328), Chi- Mei Medical Center (IRB No.: 10012-001), China Medical University Hospital (CMUH IRB No.: DMR100-IRB-214) and Kaohsiung Armed Forces General Hospital (IRB No.: 100-076). The IRBs waived the need for informed consents from source patients of the enrolled bacterial isolates because the isolates were obtained as part of routine hospital care procedures, and involved very minimal risk to the source patients; this waiver does not adversely affect the rights and welfare of the source patients.

Nonduplicate *K*. *pneumoniae* isolates collected from various sites in adult patients were tested for susceptibility to carbapenems at the participating hospitals, as part of routine laboratory procedures. Preliminary isolates that were nonsusceptible to imipenem or/and meropenem were sent to a reference laboratory at the National Health Research Institutes, Miaoli, Taiwan. After species identification was confirmed with a VITEK 2 automated system (bioMérieux, Marcy l’ Etoile, France), the isolates were stored at −70 °C until further testing. All experimental procedures were performed in accordance with specified guidelines for the use of studied isolates, and were approved by the Institutional Biosafety Committee of Chi Mei Medical Center.

### Antimicrobial Susceptibility Testing

All preliminary isolates were tested for minimum inhibitory concentrations (MICs) for β-lactam agents [including penicillin (piperacillin-tazobactam), cephalosporins (cefazolin, cefoxitin, cefuroxime, cefotaxime, ceftazidime, and cefepime), carbapenems (doripenem, ertapenem, imipenem, and meropenem), and a monobactam (aztreonam)] and non-β-lactams [including fluoroquinolones (ciprofloxacin and levofloxacin), aminoglycosides (gentamicin and amikacin), trimethoprim-sulfamethoxazole, colistin, and tigecycline]. The MIC for tigecycline was determined with the E-test (AB Biodisk, Solna, Sweden) on Mueller-Hinton media, and the MICs for other agents were determined using the broth microdilution method (Sensititre, Trek Diagnostic Systems, Cleveland, OH, USA). Isolates with MICs of >1 μg/mL for imipenem or/and meropenem were defined as CnSKP. Susceptibilities to colistin and tigecycline were determined based on the European Committee on Antimicrobial Susceptibility Testing (EUCAST) guidelines^53^, while susceptibility to other agents was based on updated guidelines from the Clinical & Laboratory Standards Institute (CLSI)^54^.

### Detection of Carbapenemase Genes

All verified CnSKP isolates were subjected to polymerase chain reaction (PCR) detection of genes encoding carbapenemases, including class A (*bla*_KPC_, *bla*_GES_, *bla*_IMI_, *bla*_NMC_, *bla*_SME_), class B (*bla*_IMP_, *bla*_VIM_, *bla*_NDM_, *bla*_GIM_, *bla*_SIM_, *bla*_SPM_), and class D (*bla*_OXA-48_), using the primers described previously ^4^. The amplicons were further sequenced to identify the molecular type. Isolates positive for the carbapenemase gene were defined as CPKP, and those that were negative were defined as non-CP-CnSKP.

### Multilocus Sequence Typing

Multilocus sequence typing (MLST) was performed on all isolates with a single carbapenemase gene according to the protocol described on the *K*. *pneumoniae* MLST website (http://www.pasteur.fr/recherche/genopole/PF8/mlst/Kpneumonia.html). MLST results were typed according to the international database created in 2005 at the Pasteur Institute in Paris, France.

### Statistical Analyses

SPSS version 17.0 (SPSS, Chicago, IL, USA) was used to perform statistical analyses. The chi-square test was applied for categorical variables, and linear-by-linear association was used to analyze trends (chi-square for trend). A p value of < 0.05 was considered statistically significant.
